# RNA-Binding Protein HuR Promotes Airway Inflammation in a House Dust Mite-Induced Allergic Asthma Model

**DOI:** 10.1089/jir.2021.0171

**Published:** 2022-01-13

**Authors:** Tomasz Herjan, Jianxin Xiao, Monika Dziendziel Kolanek

**Affiliations:** ^1^ Department of Inflammation and Immunity, Cleveland Clinic, Lerner Research Institute, Cleveland, Ohio, USA.; ^2^ Department of General Biochemistry, Faculty of Biochemistry, Biophysics, and Biotechnology, Jagiellonian University, Krakow, Poland.

**Keywords:** severe asthma, post-transcriptional control, airway inflammation, HuR

## Abstract

Mounting evidence indicates that interleukin 17 (IL-17) is critically involved in the pathogenesis of severe asthma. We have previously reported that upon IL-17 stimulation, Act1, an IL-17-receptor-complex adaptor, directly binds to its target mRNAs and utilizes other proteins, such as HuR, to upregulate mRNA stability and translation. HuR mRNA targets include multiple asthma-related genes. In this study, we have used house dust mite (HDM), a natural allergen, to test the role of HuR in the pathogenesis of allergic asthma. We found that HuR deletion in airway epithelium diminished HDM-induced lung inflammation, including neutrophil and eosinophil infiltration. While Th2 cytokines were not altered, the production of CXCL1, CXCL5 and CCL11 chemokines was significantly diminished. Airway smooth muscle (ASM) cells contribute to the pathogenesis of allergic asthma by orchestrating inflammatory and remodeling responses.

We found that IL-17 treatment of ASM cells induced translocation of HuR from nucleus to cytoplasm, where it bound directly to *Cxcl1* and *Ccl11* mRNA. Deletion of HuR in ASM cells decreased their proliferation as well as CXCL1 and CCL11 production in response to IL-17. Taken together, our findings demonstrate the importance of HuR-mediated regulation of gene expression to the pathogenesis of allergic asthma, in both airway epithelial and ASM cells.

## Introduction

Asthma has become an epidemic, affecting over 300 million people worldwide (Enilari and Sinha 2019). Airway inflammation, smooth muscle bronchoconstriction leading to airflow obstruction, and mucous hypersecretion are clinical hallmarks of asthma (Holgate and others 2015). Asthma is well known as the result of sensitization to a variety of environmental allergens and is typically associated with Th2 cytokines (IL-4, IL-5 and IL-13, IL-25, IL-33, and TSLP) and eosinophilia.

While patients with mild-to-moderate asthma (characterized by Th2 cytokine expression) usually respond well to inhaled corticosteroids, a subset of asthmatics have more severe, steroid-resistant disease characterized by neutrophilic airway inflammation and less reversible airflow obstruction (Al-Ramli and others 2009; Vazquez-Tello and others 2010; Silverpil and Lindén 2012; Fogli and others 2013; Morishima and others 2013; Chesné and others 2014; Liu and others 2017). Severe asthma is associated with a subset of T helper cells, called Th17, expressing cytokine IL-17 (also known as IL-17A) (Al-Ramli and others 2009; Park and Lee 2010; Wang and Wills-Karp 2011; Trevor and Deshane 2014).

IL-17 regulates tissue inflammatory responses, including airway inflammation, through transcriptional and post-transcriptional upregulation of proinflammatory, neutrophil-mobilizing cytokines and chemokines (Hartupee and others 2007; Sun and others 2011; Gu and others 2013). Chemokine and cytokine mRNAs are often constitutively unstable and need to be stabilized for efficient translation (Seko and others 2006; Stoecklin and others 2006; Anderson 2008; Stumpo and others 2010; Schoenberg and Maquat 2012).

Recently, we have demonstrated that Act1, an IL-17-receptor-complex adaptor, orchestrates post transcriptional branch of IL-17 signaling; Act1 directly binds and regulates messenger RNAs encoding key inflammatory proteins (Herjan and others 2018). Mechanistically, Act1 recognizes specific sequence motifs in its target mRNAs and recruits other proteins, including IKKi, TBK1, and HuR (Human antigen R), to regulate different stages of mRNA metabolism. In particular, we have shown that Act1 cooperates with HuR to upregulate target mRNA translation (Herjan and others 2013, 2018). HuR is well known as a positive regulator of both mRNA stability and translation (Peng and others 1998; Brennan and Steitz 2001; Tiedje and others 2012; Herjan and others 2013; García-Mauriño and others 2017).

Several studies have shown that HuR upregulates mRNA of cytokines and chemokines important for asthma, in various cell types, including Th2 cells, airway epithelial and airway smooth muscle (ASM) cells (Casolaro and others 2008; Fan and others 2011; Srikantan and Gorospe 2012; Atasoy and others 2014). Recently, we have shown that deletion of HuR in the airway epithelium reduced airway neutrophilia induced with intranasal IL-17 challenge (Herjan and others 2013). Despite this advance, clinically relevant allergic asthma model is necessary to fully understand the role of HuR in the pathogenesis of this multifactorial disease.

House dust mite (HDM) is a natural aeroallergen to which asthmatics are frequently sensitized. Exposure to HDM induces both Th2 and Th17-driven inflammation and results in the development of different features of asthma, including neutrophilic airway inflammation as well as airway hyperresponsiveness (Chesné and others 2015). Moreover, IL-17 has been shown to contribute to neutrophilia as well as ASM contraction observed in HDM model (Bulek and others 2019, p. 35; Chenuet and others 2017; Chesné and others 2015; Kudo and others 2012).

Therefore, in this study, we have utilized HDM allergic asthma model to examine the effect of epithelium-specific HuR knockout in asthma pathogenesis. Additionally, we assessed the impact of HuR deletion in ASM cells on IL-17 response. We found that HuR deletion in airway epithelium diminished HDM-induced pulmonary inflammation, mainly by reducing levels of neutrophil-attracting chemokines, such as CXCL1 and CXCL5, as well as an eosinophil-specific chemoattractant CCL11, but did not alter Th2 cytokines. Bronchoalveolar lavage (BAL) fluid analysis and lung histology showed significantly lower neutrophil an eosinophil infiltration upon airway epithelial-specific HuR deletion.

Mechanistically, we found that HuR deletion reduced association of *Cxcl1*, *Cxcl5*, and *Ccl11* mRNAs with actively translating polysomes. Besides epithelial cells, also ASM cells contribute to the pathogenesis of allergic asthma, by participating in inflammatory and remodeling responses (Chang and others 2012; Dragon and others 2014). We found that IL-17 treatment induced translocation of HuR from nucleus to cytoplasm in ASM cells as well as direct binding of HuR to *Cxcl1* and *Ccl11* mRNAs.

HuR deletion in primary ASM cells isolated from HuR^flox/flox^ mice, resulted in significant reduction of IL-17-induced cell proliferation as well as diminished secretion of both CXCL1 and CCL11. Mechanistically, HuR deletion in ASM cells impaired IL-17-induced association of *Cxcl1* and *Ccl11* mRNA with actively translating polysomes as well as stability of these transcripts. Taken together, our findings demonstrate significant contribution of post-transcriptional regulation of inflammatory genes mediated by HuR to the pathogenesis of allergic asthma, both in the airway epithelial cells, as well as in ASM cells.

## Materials and Methods

### Reagents

Abs against GAPDH, HuR, Histone H3, and α-Tubulin antibody were from Santa Cruz Biotechnology.; anti–pro-SP-C Ab was from Upstate. Adenoviruses encoding GFP and Cre-GFP were obtained from Vector BioLabs. For enzyme-linked immunosorbent assay (ELISA), DuoSet ELISA Development Systems (R&D Systems) was used, following the manufacturer's instructions.

### Mice

SP-C-rtTA/tetO-CRE mice, described previously (Perl and others 2009), were a kind gift from Dr. Jeffrey Whitsett. Conditional HuR-knockout (KO) embryonic stem (ES) cells and mice were generated using gene-targeting technology as described before (Herjan and others 2013). Conditional deletion of HuR in alveolar type II cells was achieved as reported before (Herjan and others 2013). All animal experiments were approved by the Institutional Animal Care and Use Committee of the Cleveland Clinic.

### HDM-induced asthma

Eight-week-old mice were sensitized subcutaneously with HDM (100 μg per mouse; Dermatophagoides farinae, Greer Laboratories) in complete Freund's adjuvant (CFA) on day 0 and then intranasally challenged with HDM (100 μg per mouse) on day 14. BAL cell counting and tissue collection were performed 24 h after the last HDM challenge.

### Histochemistry and immunohistochemistry

Hematoxylin and Eosin staining: lung tissue was fixed in 10% neutral-buffered formalin and paraffin embedding. Paraffin-embedded lung sections were stained with H&E to evaluate inflammation. For frozen sections, lungs were embedded in OCT (Tissue-Tek) and snap frozen in liquid nitrogen. Sections (10 mm) were incubated with anti-HuR (1:100) and anti–pro-SP-C (1:100). Ags were visualized following incubation with fluorescence-conjugated secondary Abs (Molecular Probes).

### Mouse ASMC isolation

Mouse smooth muscle cells were isolated as described before (Lauer and others 2009a, 2009b). Briefly, tracheas were excised, longitudinally cut, and then digested in 0.15% Pronase solution (Roche) at 4°C overnight. Next, the remaining epithelial cells were removed with a cotton swab and tracheas were cut into small pieces (∼30 per trachea). Trachea fragments were transferred to a 100-cm^2^ tissue culture dish for attachment and outgrowth of ASMCs.

### Quantitative real-time PCR

Total RNA was isolated with TRIzol reagent (Invitrogen). The cDNA was synthesized with random hexamers (Applied Biosystems) and M-MLV reverse transcriptase (Promega). Real-time PCR was performed using the SYBR Green PCR Master Mix Kit (Applied Biosystems). All gene expression results were calculated by the change in cycle threshold (ΔC_T_) method, where (ΔC_T_ = C_T_ of target gene − C_T_ of either *Actb* (encoding β-actin) or *Gapdh*, and are presented as 2^(–ΔC_T_). The primers used for qPCR are listed in Table 1.

**Table 1.** Primers for Real-Time Quantitative Polymerase Chain Reaction

mCXCL1	F: CTGGCCACAGGGGCGCCTATC R: GGACACCTTTTAGCATCTTT
mCXCL5	F:GTTCCATCTCGCCATTCATGC R:GCGGCTATGACTGAGGAAGG
mIL-5	F: CTCACCGAGCTCTGTTGACAAG R: CCAATGCATAGCTGGTGATTTTTAT
mIL-13	F: TGACCAACATCTCCAATTGCA R:TTGTTATAAAGTGGGCTACTTCGATTT
mβ-actin	F: GGTCATCACTATTGGCAACG R: ACGGATGTCAACGTCACACT
mCCL11	F: GAATCACCAACAACAGATGCAC R:ATCCTGGACCCACTTCTTCTT
mIL-17	F: CTCCACCGCAATGAAGAC R: CTTTCCCTCCGCATTGAC

### RNA-binding assays RIP

For HuR-RNA immunoprecipitation 10 × 10^6^ cells were trypsinized, washed twice, and resuspended in 10 mL ice-cold PBS. Cells were fixed in 0.1% formaldehyde for 15 min at room temperature, whereupon the crosslinking reaction was stopped with glycine (pH 7; 0.25 M). The cells were then washed twice with ice-cold PBS, resuspended in 2 mL RIPA buffer (50 mM Tris-HCl [pH 7.5], 1% Nonidet P-40, 0.5% sodium deoxycholate, 0.05% SDS, 1 mM EDTA, 150 mM NaCl, and proteinase inhibitors), and sonicated. The lysate was centrifuged (15 min, 4°C, 16,000 *g*), and 1 mL each of supernatant was immunoprecipitated overnight at 4°C, using Dynabeads (Invitrogen) preincubated with 20 μg anti-M2 or anti-IgG Ab. The beads were washed 5 times with 1 mL RIPA buffer and resuspended in 150 μL elution buffer (50 mM Tris-Cl [pH 7], 5 mM EDTA, 10 mM DTT, 1% SDS).

Crosslinking was reversed by incubation at 70°C for 45 min, RNA was purified from immunoprecipitates with TRIzol (Invitrogen) according to the manufacturer's instructions and treated with RNase-free DNase. The cDNAs were synthesized and 10% (2 mL) of the reverse transcriptase product was subjected to quantitative real-time PCR. Primers used for quantitative real-time PCR are listed in Table 1.

### Polysomal fractionation analysis

A total of 2 × 10^8^ ASM cells was left untreated or stimulated with IL-17A (50 ng/mL) for 2 h. Cytoplasmic extracts were carefully layered over 10%–50% linear sucrose gradients in polysome buffer (10 mM HEPES [pH 7.5], 100 mM KCl, 2.5 mM MgCl_2_, 1 mM DTT, 50 U recombinant RNasin (Promega), and 0.1% IGEPAL CA-630 (Sigma) and centrifuged at 17,000 rpm in a Beckman SW32.1 Ti rotor for 4 h at 4°C. Gradients were fractioned using an ISCO gradient fractionation system equipped with a UA-6 detector. Light ribonucleoprotein (RNP) fractions, 40S, 60S, and 80S, and heavy polysome fractions were monitored by the continuous UV absorption profile at A254, and 9 tubes of 750 mL fractions were collected.

The fractions representing light RNP and free ribosomes were used to prepare the translation-inactive pool of proteins and mRNAs, and the fractions representing heavy polysomes were used to isolate the translation-active proteins and mRNAs. One tenth of each fraction was used for Western blot analysis; one fifth of each fraction was used for RNA isolation by extraction with TRIzol. For lung tissue, protocol was modified as described in Del Prete and others (2007).

In short, lung tissue (30 mg/sample) was snap frozen under liquid nitrogen. Next, tissue was pulverized under liquid nitrogen and resuspended in lysis buffer (10 mM Tris-HCl at pH 8.0, 140 mM NaCl, 1.5 mM MgCl_2_, 0.5% Nonidet-P40, 20 mM, dithiothreitol, 500 U/mL RNAsin, and 0.5% [w/v] deoxycholate). Intact nuclei were pelleted through centrifugation at 4°C, for 10 s at 12,000 *g*. Supernatant was supplemented with 500 mL of extraction buffer (0.2 M Tris-HCl at pH 7.5, 0.3 M NaCl), 150 mg/mL cycloheximide, 650 mg/mL heparin, and 10 mM phenylmethylsulfonyl fluoride) and centrifuged (12,000 *g*, 5 min, at 4°C) to remove mitochondria and membranous debris.

The supernatant was layered onto a sucrose gradient and fractions were used to prepare proteins and mRNAs as described above. Before reverse transcription, RNA was precipitated with 2 M LiCl on ice at 4°C overnight, washed twice with 70% ETOH, and resuspended in RNAse-free water.

### Subcellular fractionation

Confluent cells in 15-cm plates, untreated or treated with IL-17 (50 ng/mL) for various times, were resuspended in 1 mL ice-cold hypotonic buffer (10 mM HEPES [pH 7.4], 1.5 mM MgCl_2_, 10 mM KCl, 0.2 mM PMSF, and 0.5 mM DTT) and homogenized on ice with 45 strokes of a Dounce homogenizer. Unlysed cells, nuclei, and cell debris were pelleted by centrifugation at 1,000 *g* for 5 min 5 times. Soluble (supernatant, S100) fractions were generated by centrifugation at 100,000 *g* for 1 h.

### Adenoviral infection

Primary ASM cells were divided into 60-mm dishes and infected by exposing them to media containing 2 × 10^5^ infectious units/plaque formation units of adenovirus/mL overnight.

### Statistical analyses

Statistical analyses were applied to biologically independent samples (separate plates of cells or mice) from every single experiment. Experiments were repeated at least 3 times. For all RT-PCR and ELISA analyses, at least 3 biological replicates (separate plates of cells) were used. Comparisons between 2 groups were analyzed by 2-tailed Student's *t* tests. All bar graphs show mean and SD as indicated in each legend. GraphPad Prism 7 was used for data analysis and representation.

## Results

### HuR deletion in airway epithelial cells diminishes HDM-induced neutrophil and eosinophil airway infiltration

Severe asthma is associated with Th17 cells, expressing proinflammatory cytokine IL-17. While IL-17 activates target gene transcription, it primarily acts at the post-transcriptional level. Messenger RNAs encoding inflammatory gene products, including cytokines and chemokines, are often inherently unstable, and need to be stabilized for efficient translation. We have shown that Act1 directly binds specific mRNA sequence motifs and utilizes other proteins such as HuR to enhance stability and translation of mRNA (Herjan and others 2018).

Recently, we have also shown that deletion of HuR in the airway epithelium reduced airway neutrophilia that was induced with intranasal IL-17 challenge (Herjan and others 2013). Despite that progress, clinically relevant allergic asthma model is necessary to elucidate the role of HuR in this multifactorial disease. In this study, we used HDM, a natural aeroallergen to which asthmatics are frequently sensitized, to examine the effect of epithelium-specific HuR knockout in the pathogenesis of asthma.

First, we conditionally depleted HuR in airway epithelial cells as previously described (Herjan and others 2013); in short, we used the SFTPC (Surfactant Protein C or SP-C) gene promoter to generate the SP-C-rtTA/(tetO)_7_CMV-Cre–transgenic mice that allow doxycycline-induced expression of Cre recombinase in the distal lung respiratory epithelium. Next, we bred HuR^flox/flox^ mice onto SP-C-rtTA/(tetO)7CMV-Cre to generate conditional distal lung-specific HuR-deficient mice [SP-C-rtTA(tetO)7CMV-Cre-HuR^flox/flox^] referred to as “HuR KO” in this study. The ([SP-C-rtTA(tetO)7CMV-Cre-HuR^flox/wt^] mice, referred to as “HuR WT,” were used as control.

**FIG. 1.**
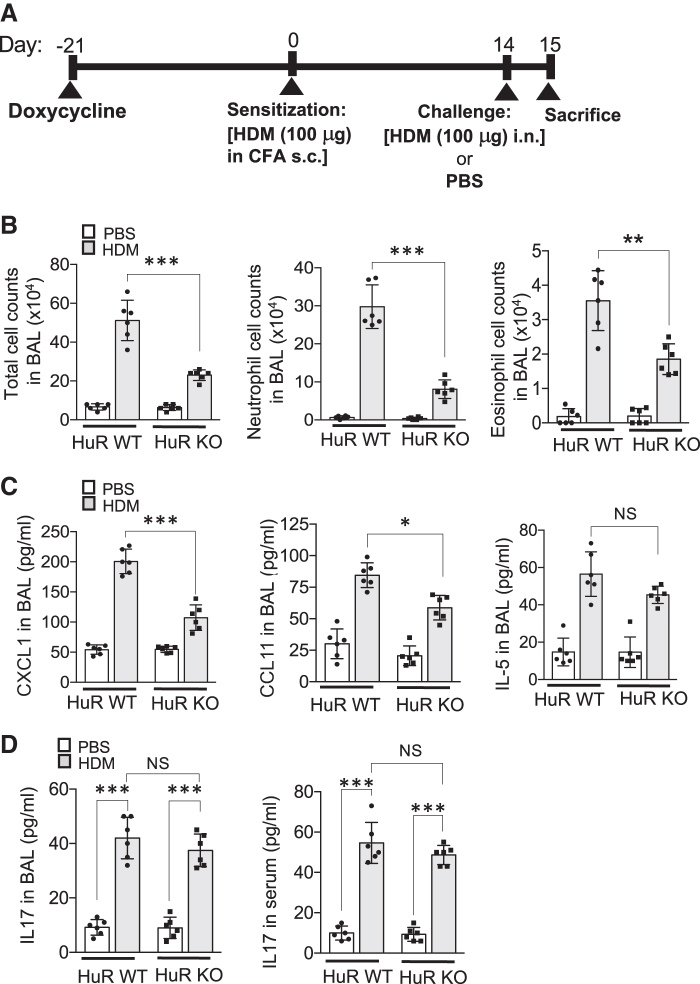
HuR deletion in airway epithelium reduces HDM-induced neutrophil and eosinophil infiltration. **(A)** Timeline of HDM-induced allergic airways inflammation: HuR ^flox/wt^ (HuR WT) and HuR ^flox/flox^ (HuR KO) mice expressing SP-CrtTA/tetO-CRE (*n* = 6/group) were administered doxycycline 3 weeks before sensitization with HDM in complete Freund's adjuvant. Fourteen days after sensitization, mice were challenged either with HDM or PBS and sacrificed 24 h later. **(B)** Total, neutrophil, and eosinophil cell numbers in the BAL of the indicated mice, treated as described in **(A)**, were manually counted (*n* = 6 mice/group). **(C)** ELISA of CXCL1, CCL11, and IL-5 in BAL fluid from indicated mice, treated as described in **(A)** (*n* = 6 mice/group). **(D)** The BAL fluid and serum levels of IL-17 in the indicated mice, treated as described in **(A)** (*n* = 6 mice/group), were measured by ELISA. Throughout figure, data represent mean and SD of biological replicates. *P < 0.05, **P < 0.01, and ***P < 0.001; NS, not significant. All data are representative of 3 independent experiments. Scale bars, 100 μm. BAL, bronchoalveolar lavage; ELISA, enzyme-linked immunosorbent assay; HDM, house dust mite.

Subsequently, these HuR KO and control HuR WT mice were administered doxycycline for 3 weeks and then were sensitized subcutaneously with HDM in CFA. Fourteen days after sensitization mice were challenged either with HDM or PBS and sacrificed 24 h later (Fig. 1A and Supplementary Fig. S1).

Next, we analyzed cellular components of the BAL fluid and found that HuR depletion attenuated neutrophilia as well as decreased the number of infiltrating eosinophils (Fig. 1B and Supplementary Fig. S2). ELISA was used to measure cytokine levels in BAL. CXCL1, a key neutrophil chemoattractant was strongly reduced in HuR KO as compared with HuR WT (Fig. 1C). Mouse eotaxin (CCL11) is an eosinophil-specific chemoattractant that has been shown to be secreted by airway epithelial cells in various asthma models. We found that HuR depletion decreased CCL11 protein level in BAL form HuR KO mice. TH2 cells orchestrate the inflammation in asthma through secretion of various cytokines. The level of IL-5, a typical Th2-derived cytokine, was induced by HDM challenge but not significantly affected by HuR depletion in airway epithelium (Fig. 1C).

Multiple studies have found a strong correlation between levels of serum IL-17 and asthma severity (Agache and others 2010; Chesné and others 2014). Serum IL-17 concentrations in severe asthma were significantly increased as compared with moderate forms of the disease. In this study, we found that HDM challenge increased serum and BAL fluid IL-17 levels to a similar degree in both HuR WT and KO mice (Fig. 1D).

### Deletion of HuR in the airway epithelium attenuates HDM-induced lung inflammation through inhibition of CXCL1, CXCL5 and CCL11 production

**FIG. 2.**
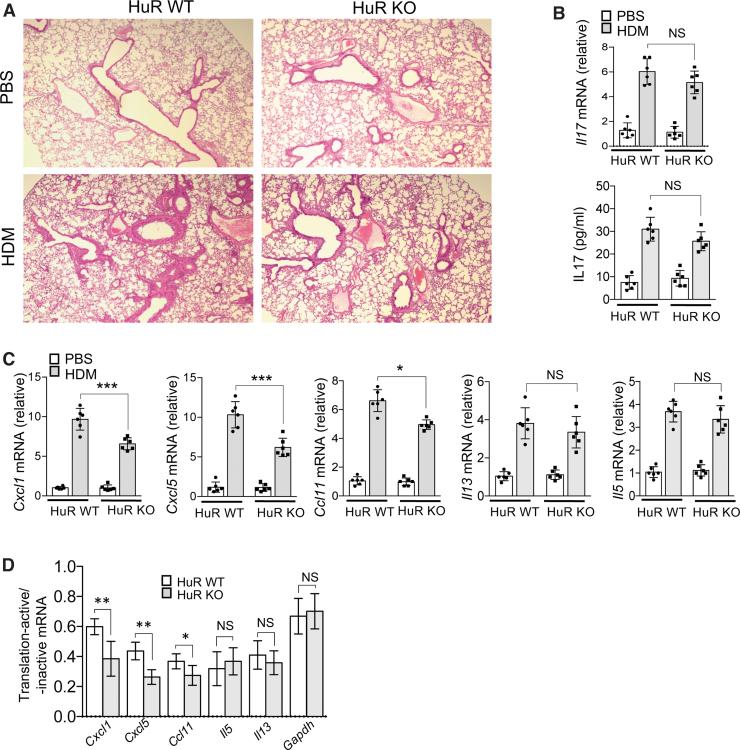
HuR depletion in airway epithelial cells diminishes HDM-induced pulmonary inflammation. **(A)** Lung tissue from indicated mice, treated as described in Fig. 1A (*n* = 6/group), was subjected to paraffin sectioning and Hematoxylin–Eosin staining. **(B)** Real-time PCR analysis of *IL17* mRNA (*upper panel*) and ELISA analysis of IL-17 protein (*lower panel*) in the lung tissue of the indicated mice, treated as described in Fig. 1A (*n* = 6/group); graphs show mean fold induction over PBS-treated group. **(C)** The relative abundances of mRNAs isolated from lung tissue of the indicated mice, treated as described in Fig. 1A (*n* = 6/group); graphs show mean fold induction over PBS-treated group). **(D)** Lung extracts from indicated mice, treated as described in Fig. 1A (*n* = 6/group), were fractionated through a 10%–50% sucrose gradient and separated into translation-active pools and translation-inactive pools as described in the Materials and Methods section. Indicated mRNAs isolated from translation-active and translation-inactive pools were analyzed by RT-PCR and normalized to β-actin. Graph shows the ratios of mRNAs from translation-active/inactive pools. Throughout the figure, data represent mean and SD of biological replicates. *P < 0.05, **P < 0.01, and ***P < 0.001; NS, not significant. All data are representative of 3 independent experiments. Scale bars, 100 μm. Color images are available online.

Subsequently, histological analysis of lung tissue revealed significantly less lung inflammation in HuR KO mice (Fig. 2A). It has been shown that IL-17 plays an important role in a mouse model of asthma induced by HDM (Chesné and others 2015). We indeed observed markedly increased IL-17 mRNA and protein levels in total lung tissue from HDM-treated mice, although it was not altered by HuR depletion in airway epithelium (Fig. 2B). Interestingly, mRNA expression levels of proinflammatory chemokines, *Cxcl1 and Cxcl5*, were significantly lower in total lung tissue from HuR KO mice; *Ccl11 mRNA* level was also reduced but to a lesser degree (Fig. 2C). Both *IL-5* and *IL-13* mRNAs were not significantly altered by epithelial HuR depletion.

Recently, we have shown that HuR plays a critical role in IL-17-induced translation of certain mRNAs; IL-17 treatment induced coshift of Act1-HuR-mRNA complexes to polysomes (Herjan and others 2013, 2018). Therefore, in this study, we analyzed polysome-bound mRNA in total lung tissue from HDM-challenged HuR WT and KO mice, by performing polysomal fractionation. We found that while HuR depletion significantly reduced the association of *Cxcl1*, *Cxcl5*, and *Ccl11* mRNA with actively translating polysomes, *Il-13* or *Il-5* mRNAs were unaffected (Fig. 2D).

### HuR promotes ASM cell proliferation as well as CXCL1 and CCL11 production in response to IL-17 treatment

Airway remodeling is a critical feature of asthma, and it is linked to extracellular matrix deposition and enhanced ASM cell proliferation. Interestingly, IL-17 has been shown to directly affect ASM cell proliferation and contraction (Kudo and others 2012; Bulek and others 2019). Since we found increased IL-17 concentrations in BAL, lungs, and serum of HDM-challenged mice, we next assessed the effect of HuR deletion in primary ASM cells on their response to IL-17 treatment.

**FIG. 3.**
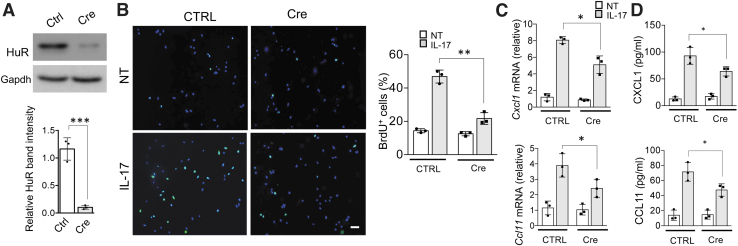
HuR deletion impairs IL-17-induced ASM cell proliferation and cytokine production. **(A)** *Upper panel*: representative western blot analysis of HuR in lysates from primary mouse ASM cells infected with a GFP-encoding adenovirus (Control) or Cre-GFP-encoding adenovirus (Cre); *lower panel*: western blots were quantified by densitometry using ImageJ. **(B)** *Right*: representative images of Serum-starved primary ASM cells either left untreated or treated with IL-17 (50 ng/mL) for 24 h; BrdU was added during the last 3 h. *Left*: Bar graph shows the mean and SD of percentages (*n* = 3 independent plates) of BrdU^+^ cells per 10 × magnification field. **(C, D)** ASM cells infected with a GFP-encoding adenovirus (Control) or Cre-GFP-encoding adenovirus (Cre) were left untreated or stimulated with IL-17A for 12 h. The mRNA and protein levels were then analyzed by RT-PCR **(C)** and ELISA **(D)**, respectively (*n* = 3 independent plates of cells); bar graphs show mean and SD of independent plates of cells. *P < 0.05, **P < 0.01, and ***P < 0.001 (WT vs. KO); NS, not significant. All data are representative of 3 independent experiments. Scale bars, 100 μm. ASM, airway smooth muscle. Color images are available online.

First, we focused on IL-17-induced cell proliferation and cytokine production. We isolated primary airway muscle cells from HuR^flox/flox^ mice and infected these cells with adenovirus encoding Cre-recombinase to delete HuR (Fig. 3A). Then, we assessed cell proliferation using a thymidine analog, bromodeoxyuridine (BrdU), which is incorporated into DNA during S phase of the cell cycle. Both control- and Cre adenovirus-infected cells were serum starved and then stimulated with IL-17 for 24 h; BrdU was added during the last 3 h.

Interestingly, while IL-17 treatment significantly increased BrdU incorporation, it was strongly attenuated by HuR depletion (Fig. 3B). ASM cells contribute to asthma also as a source of inflammatory mediators. Indeed, we found that treatment of ASM cells with IL-17 for 12 h robustly induced *Cxcl1* and *Ccl11* mRNA expression as well as secretion of these proteins to the cell culture supernatant (Fig. 3C, D). However, depletion of HuR strongly reduced *Cxcl1* and *Ccl11* mRNA and protein levels (Fig. 3C, D).

### Upon IL-17 treatment HuR translocates from nucleus to cytoplasm where it directly regulates Cxcl1 and Ccl11 mRNAs

**FIG. 4.**
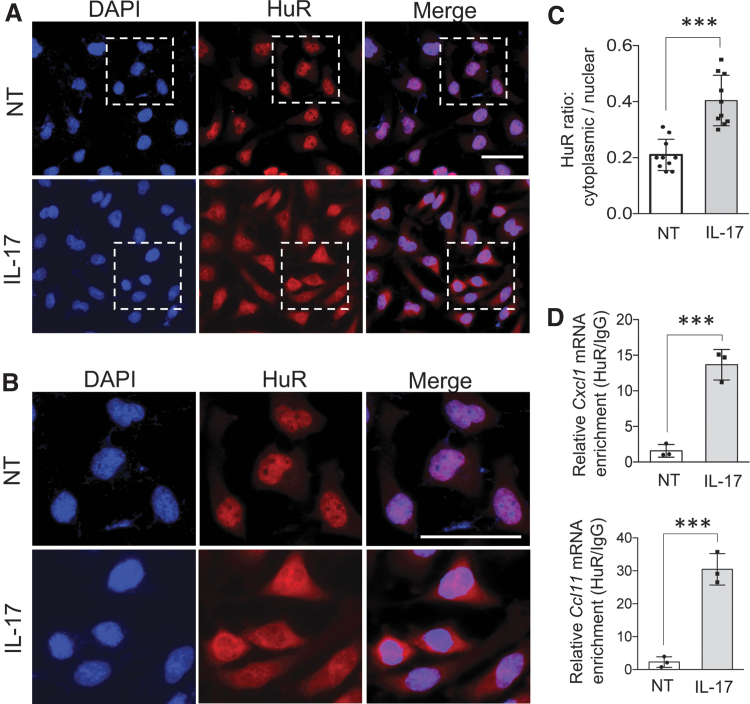
Upon Il-17 treatment, HuR translocates to cytoplasm and directly binds to *Cxcl1* and *Ccl11 mRNA.* **(A)** Primary ASM cells either left untreated or treated with IL-17 (50 ng/mL) for 8 h were fixed and stained for HuR (*red*), and DAPI (*blue*) stained the nucleus. **(B)** Zoomed-in region highlighted by *white dashed box* in **(A)**. **(C)** Bar graph showing cytoplasmic to nuclear ratio of HuR in cells described in **(A)**, quantified using ImageJ (mean and SD of 10 independent plates of cells). **(D)** ASM cells either left untreated or treated with IL-17 (50 ng/mL) for 2 h were subjected to RNA immunoprecipitation with anti-HuR or anti-IgG, and RT-PCR analyses (*n* = 3 independent plates of cells) of the indicated mRNAs. Relative values normalized against IgG control are shown (mean and SD of independent plates of cells). ***P < 0.001; NS, not significant. All data are representative of 3 independent experiments. Scale bars, 50 μm. Color images are available online.

HuR's ability to regulate stability and translation of mRNAs has been linked to its translocation from nucleus to cytoplasm (Fan and Steitz 1998; Wang and others 2006; Kim and Gorospe 2008). We indeed found that IL-17 treatment of ASM cells for 8 h resulted in increased accumulation of HuR in the cytoplasm (Fig. 4A–C and Supplementary Fig. S3). Moreover, HuR-RNA immunoprecipitation (RIP) experiment revealed that IL-17 stimulation induced association of HuR with *Cxcl1* and *Ccl11* mRNAs, which suggests that HuR directly regulates these transcripts (Fig. 4D).

**FIG. 5.**
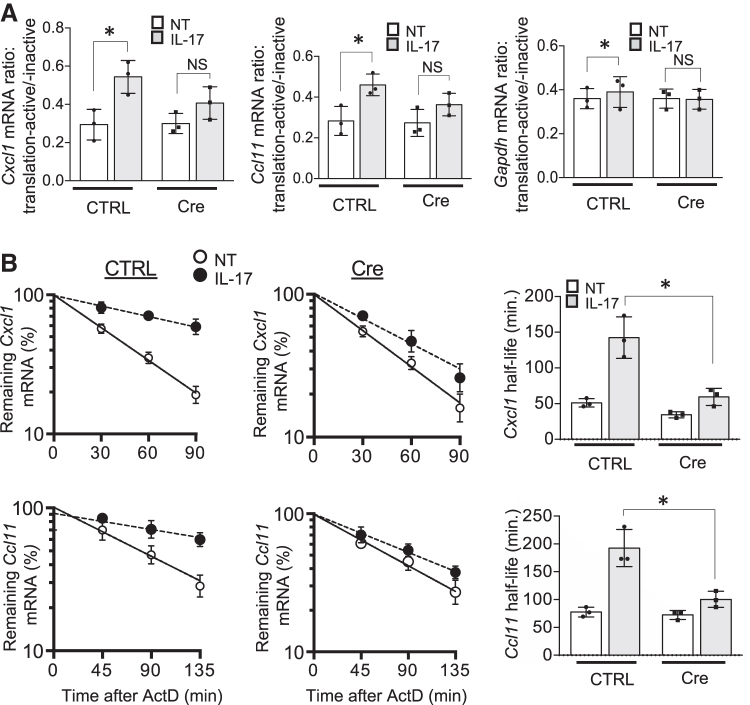
HuR enhances *Cxcl1* and *Ccl11* mRNA translation and stability in response to IL-17 stimulation. **(A)** Cytoplasmic extracts of ASM cells infected as in Fig. 3A, either left untreated (NT) or treated with IL-17 (50 ng/mL) for 2 h were fractionated through a 10%–50% sucrose gradient, as described in the Materials and Methods section; bar graph represents the ratios of indicated mRNAs isolated from translation-active and translation-inactive fractions (mean and SD of 3 independent plates of cells). **(B)** Real-time PCR analysis of *Cxcl1* and *Ccl11* in ASM cells, infected as in Fig. 3A, which were pretreated for 2 h with TNF (10 ng/mL), followed by actinomycin D (5 mg/mL) alone (NT) or in combination with IL-17 (50 ng/mL) for the indicated time (mean and SD of 3 independent plates of cells). The results are presented as decay over time (*left panels*) and as half-life (*right panels*). *P < 0.05; NS, not significant. All data are representative of 3 independent experiments.

Therefore, we assessed the impact of HuR deletion on IL-17-driven stabilization and translation of the *Cxcl1* and *Ccl11* mRNAs. First, we separated Control- and Cre adenovirus-infected ASM cell lysates that were either untreated or treated with IL-17, into translation-inactive free ribosome and translation-active polysome fractions. Next, we isolated RNA from these fractions and found that HuR deletion significantly diminished IL-17-induced association of *Cxcl1* and *Ccl11* mRNAs with polysomes (Fig. 5A).

We then measured the impact of HuR deficiency on IL-17-induced mRNA stabilization. ASM cells were first pretreated for 0.5 h with TNF-α (to induce inflammatory gene transcription) followed by treatment with actinomycin D (to block transcription) along with IL-17 (for mRNA stabilization). Both *Cxcl1* and *Ccl11* mRNA was induced to a similar extent in Control- and Cre adenovirus-infected ASM cells after the initial TNF-α pretreatment, but decayed more rapidly in HuR-deficient ASM cells (Fig. 5B). These results indicate that HuR is necessary for IL-17-mediated stabilization of *Cxcl1* and *Ccl11* mRNAs.

## Discussion

Post-transcriptional control is increasingly recognized as a major mechanism regulating genes expressing short-lived mRNAs (such as growth factors, cytokines, and chemokines), and thus as an important contributor to various diseases, including asthma. Mounting evidence indicates that IL-17 is critically involved in the pathogenesis of a severe, steroid-resistant asthma. Interestingly, while IL-17 alone is a relatively weak inducer of gene expression, in cooperation with other cytokines, such as TNF-α, it generates a strong response, mainly through upregulation of mRNA stability and translation (Hartupee and others 2007).

Previously we found that Act1, an IL-17-receptor-complex adaptor, directly binds specific mRNA sequence motifs and utilizes other proteins such as HuR to regulate mRNA metabolism (Herjan and others 2018). We have also demonstrated that deletion of HuR in the airway epithelium diminished airway neutrophilia induced with intranasal IL-17 challenge (Herjan and others 2013), which provided initial evidence for importance of the post-transcriptional control mediated by IL-17/Act1/HuR axis in asthma.

HDM model used in this study utilizes natural aeroallergen and induces both Th2 and Th17-driven inflammation. We found that epithelial-specific HuR deletion was able to diminish inflammation in this multifactorial asthma model. This fact implicates not only the importance of HuR-mediated regulation of gene expression but also the central role of airway epithelium in the pathogenesis of allergic asthma.

Another nonimmune cell type involved in asthma pathogenesis is ASM cells. The role of ASM cells as a source of inflammatory mediators has been recently recognized. HuR deletion in ASM cells not only attenuated cytokine expression, but it also reduced IL-17-mediated ASM cell proliferation, which implicates involvement of HuR in airway hyper-responsiveness and remodeling. As a future study, it will be important to determine mechanism of HuR-mediated regulation of ASM cell proliferation. Interestingly, HuR has been shown before to regulate cell proliferation through stabilization of mRNAs encoding cell cycle regulators: cyclin A and cyclin B7 (Wang and others 2000). Moreover, HuR targets include mRNAs of proliferation-associated genes, such as c-fos (Chen and others 2002).

Subsequently, we found that IL-17 treatment induces translocation of HuR to cytoplasm, association of HuR with *Cxcl1* and *Ccl11* mRNAs and upregulation of their stability and translation. All these observations further support the importance of HuR for the response of ASM cells to IL-17 treatment. In future studies, it will be important to examine the effect of HuR deletion in ASM cells using animal asthma model, such as HDM.

Taken together, our findings suggest that post-transcriptional regulation mediated by HuR in both airway epithelial cells and ASM cells has an important impact on the pathogenesis of allergic asthma.

## Supplementary Material

Supplemental data

Supplemental data

Supplemental data
