# Supplementary material for: RNA-Binding Protein HuR Promotes Airway Inflammation in a House Dust Mite-Induced Allergic Asthma Model
Source: J Interferon Cytokine Res. 2022 Jan 13;42(1):29–38. doi: 10.1089/jir.2021.0171 (PMC8787712; doi:10.1089/jir.2021.0171)
Supplement: Supplemental data [file Suppl_Figure1.pdf]

Supplementary Figure 1.

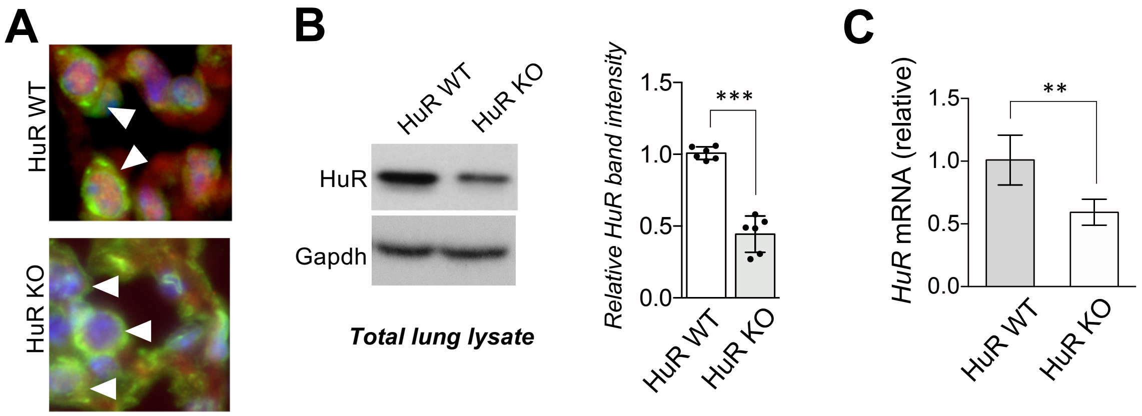

Supplementary Figure 1. A. Paraffin-embedded sections of SPCrtTA/tetO-CRE/ HuR$^{flox/flox}$ (HuR-KO) and SPCrtTA /tetO-CRE/ HuR$^{flox/wt}$ (HuR WT) lungs were stained for HuR (red) and pro-SP-C (green), DAPI (blue) stained the nucleus. A subset of alveolar type II cells (pro–SP-C) is indicated with arrowheads.

B. Left: western blot analysis of HuR and Gapdh in lung homogenates from HuR $^{flox/wt}$ (HuR WT) and HuR $^{flox/flox}$ (HuR KO) mice expressing SP-CrtTA/tetO-CRE (n = 6/group), treated as in Fig. 1A, right: western blots were quantified by densitometry using ImageJ. **C**. Lung tissue from mice in (**B**) was snap frozen in liquid nitrogen followed by RNA extraction and real-time RT-PCR analysis. Throughout figure, data represent mean and s.d of biological replicates. *P < 0.05, **P < 0.01 and ***P < 0.001 by two-tailed Student t test; NS, not significant.
