# Supplementary material for: RNA-Binding Protein HuR Promotes Airway Inflammation in a House Dust Mite-Induced Allergic Asthma Model
Source: J Interferon Cytokine Res. 2022 Jan 13;42(1):29–38. doi: 10.1089/jir.2021.0171 (PMC8787712; doi:10.1089/jir.2021.0171)
Supplement: Supplemental data [file Suppl_Figure2.pdf]

## Supplementary Figure 2.

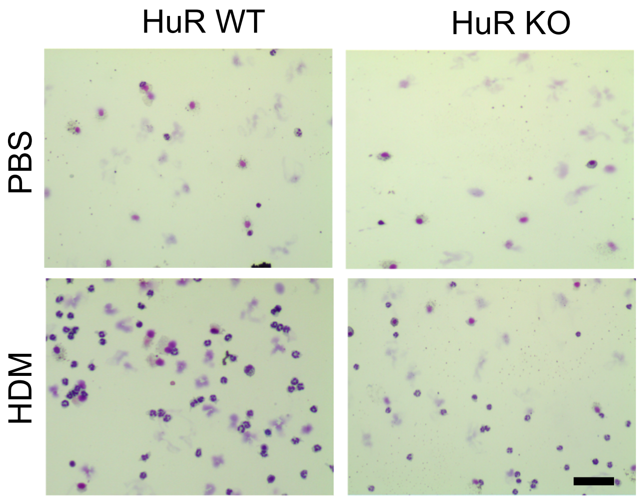


**Supplementary Figure 2**. Representative BAL fluid cytospins from the indicated mice, treated as described in Fig. 1 A (*n* = 6 mice/ group), subjected to HEMA3 staining.
