# Supplementary material for: RNA-Binding Protein HuR Promotes Airway Inflammation in a House Dust Mite-Induced Allergic Asthma Model
Source: J Interferon Cytokine Res. 2022 Jan 13;42(1):29–38. doi: 10.1089/jir.2021.0171 (PMC8787712; doi:10.1089/jir.2021.0171)
Supplement: Supplemental data [file Suppl_Figure3.pdf]

Supplementary Figure 3.

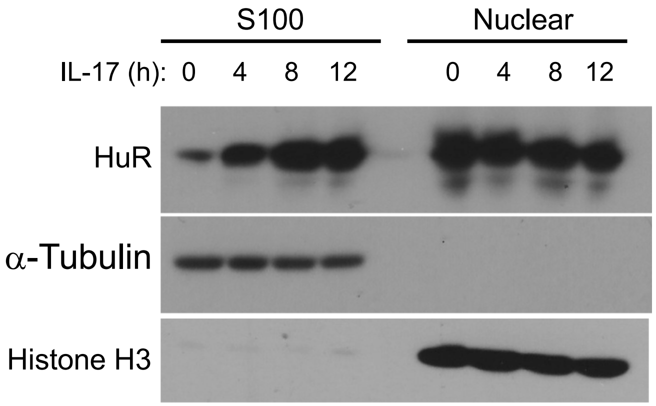

Supplementary Figure 3. Cell lysates from primary ASM cells either left untreated or treated with IL-17 (50 ng/ml) for indicated time were fractionated into cytosolic (S100), and nuclear fractions, as described in *Materials and Methods*, followed by Western blot analyses of α-Tubulin, HuR, and Histone H3.
